# Bhartṛhari on Linguistic Universals and the Problem of Regress

**DOI:** 10.1007/s11407-025-09411-x

**Published:** 2025-09-15

**Authors:** Marco Ferrante

**Affiliations:** Institute for the Cultural and Intellectual History of Asia, Austrian Academy of Sciences, Dominikanerbastei 16, 1010 Vienna, Austria

**Keywords:** Bhartṛhari, Metaphysics, Vaiśeṣika, Universals, Ontology, Indian philosophy

## Abstract

This article explores the philosophical and linguistic inquiries of Bhartṛhari, a renowned grammarian and philosopher who lived between 460 and 510 CE. Focusing on his seminal work, the *Vākyapadīya*, the article examines Bhartṛhari’s investigation into the problem of universals. It begins by providing an overview of the debates surrounding universals in both Euro-American and Indian philosophies. The article then delves into Bhartṛhari’s unique distinction between linguistic and ontic universals, analyzing his arguments and discussing how his concepts address a common criticism faced by universal realists—the alleged vicious regress of universals.

## Introduction

Universals are one possible solution to the philosophical problem of attribute agreement: how can we explain that objects appear to possess shared qualities? Do these qualities represent abstract, universal properties manifested by specific objects? Or is attribute agreement a result of a conceptual framework we employ to make sense of our experiences? In Euro-American philosophy, these two approaches broadly correspond to the well-known dichotomy between realism, which posits the existence of universal properties, and nominalism, which rejects it. Over time, numerous metaphysicians have shaped, refined, and modified these two foundational theories to establish their superiority over the opposing perspective.

Notably, this issue also held central importance in the philosophical traditions of premodern India, where a debate resembling that between realism and nominalism took place. This article explores how the linguist and philosopher Bhartṛhari (fifth century CE) addressed the problem of universals in his masterwork, the *Vākyapadīya*. Specifically, the focus of this contribution lies in Bhartṛhari’s crucial differentiation between linguistic universals (*śabdajāti*) and ontic universals (*arthajāti*). In the subsequent discussion, I will argue that this distinction demonstrates a sophisticated approach to the problem and presents an ingenious solution to a concern frequently faced by realism: the potential trap of an infinite regress of universals.

## On the Classification of Objects: The Function of Universals

Realists propose an explanation for attribute agreement by positing the existence of universals. According to this perspective, when objects *x*, *y*, and *z* share a common attribute, there exists a universal entity F-ness and a relation R such that *x*, *y*, and *z* stand in the relation R to F-ness. Universals are independent and repeatable entities not confined to specific spatiotemporal locations, while *x*, *y*, and *z* are particular objects that instantiate or exemplify the universal property F-ness. In other words, a particular object is “red” because it exemplifies or instantiates the universal property “redness.” In Euro-American philosophy, this explanatory model finds its historical roots in Plato’s work. In the *Parmenides* (130e–131a), Plato wrote: “There exist certain forms, of which these other things come to partake and so to be called after their names; by coming to partake of likeness or largeness or beauty or justice, they become like or large or beautiful or just” (1961: 925). Subsequent realist positions have been significantly influenced by Plato’s formulation, leading to the designation of this model as the “Platonic scheme.”

Realists argue that acknowledging the existence of universals offers an elegant and economical solution to various ontological problems. One notable problem concerns the truth of subject-predicate sentences. From a realist standpoint, a statement like “Rāma is honest” is true because an individual *x* (Rāma) exemplifies the specific quality or property F-ness, namely, “being honest” or “honesty.” Importantly, realists assert a complete correspondence between the linguistic statement “Rāma is honest” and the ontological entities they refer to. Just as the term “Rāma” designates a particular ontic entity, the term “honest” designates a specific, repeatable, independent, and universal property: “honesty.” Consequently, the particular object *x* and the universal property F-ness possess the same ontological status within the realist framework.^1^

As expected, nominalists raise objections to this model, with a key concern being the potential regress of universals.^2^ Let us reconsider the sentence “Rāma is honest” and generalize it as follows:

(1) *x* is F.

For a realist, the truth of (1) rests on the idea that:

(2) *x* exemplifies F-ness.

However, (2) can be rewritten as “*x* is an exemplification of F-ness,” which clearly is another subject-predicate sentence. Thus, following the realist scheme, (2) is deemed valid solely based on:

(3) *x* exemplifies [an exemplification of F-ness].

This analysis reveals a regress, as the truth of (3) would then depend on:

(4) *x* exemplifies [an exemplification of an exemplification of F-ness].

If this regress turns out to be vicious, one might argue that the theory of universals fails to provide a satisfactory explanation for attribute agreement. The challenges realists face extend even further, as the regress criticism can be applied to the notion of relation itself. In the Platonic scheme, a relation of exemplification is necessary for a universal to be instantiated by a particular. However, according to this view, relations themselves are universals, entities that exist independently of their relata. This perspective raises the issue that establishing a relation R between objects A and B would require invoking another relation R_2_, which in turn would necessitate yet another relation R_3_, and so on, leading to an infinite regress.^3^

Over time, realists have developed various strategies to address the challenge of regress in their theory. One common approach is outright dismissing the criticism, arguing that their theory adequately explains attribute agreement at the first level and is not intended to account for higher-level cases. In other words, while regress may exist, realists argue it is not inherently problematic or vicious. Alternatively, other theorists propose restrictions on specific aspects of the scheme, such as exemplification. According to this perspective, exemplification is not regarded as a relation, and the connection between particular objects and universals is better described using neutral terms like “tie” or “nexus.”

Finally, it is worth noting that abandoning universals and adopting a nominalist perspective does not resolve the regress problem either. Nominalists explain attribute agreement by positing that a subject-predicate sentence like “*x* is F” can be accounted for by identifying a particular condition C, such that “*x* is F” is true whenever C is fulfilled. However, by doing so, a new subject-predicate sentence emerges (“*x* is such that C is fulfilled”), necessitating another condition, C1, to be true, and so on.

## Universals in the Indian Philosophical Tradition

In Indian philosophy, the debate on universals follows a similar trajectory. Realism, broadly intended, is the dominant position espoused by thinkers from various backgrounds, primarily belonging to the Brāhmaṇical milieu.^4^ These philosophers uphold a viewpoint akin to the one we have just explored: universals possess an existence independent of particulars, and even if all particulars were hypothetically destroyed, the existence of their corresponding universals would remain unaffected. It is nonetheless crucial to note that, despite variations, all Indian realists start from a premise that differs somewhat from that of their Euro-American counterparts. They perceive the ontological problem of universals as intimately linked to the semantic issue of word-meaning. Consequently, the problem of attribute agreement is not necessarily expressed in ontological terms, such as why different red objects share the quality of “redness” but rather as the question of how one linguistic unit can refer to multiple particulars. In practical terms, this translates into identifying the reason for using the same sequence of sounds, like “cow,” to denote the many individual cows encountered in ordinary experience. Universal realists typically argue that a common generic property (*jāti*, *sāmānya*), such as “cowness,” is instantiated in all individual cows. On the other hand, other thinkers contend that the word “cow” primarily designates an individual entity or substance (*vyakti*, *dravya*). This continuous interplay between semantic and ontological aspects makes it challenging to reconstruct the contours of the debate on universals in Indian philosophical literature. However, available information seems to suggest that the discussion of the semantic dimension precedes the ontological one.^5^ Within the Pāṇinian grammatical tradition, already Kātyāyana (third century BCE) mentions the names of two ancient grammarians, Vyāḍi and Vājapyāyana, who held divergent opinions on the nature of word-meaning. Vyāḍi believed that a word always denotes a particular substance, while Vājapyāyana argued for a generic property.^6^ These names eventually became representative of two alternative approaches to explaining word-meaning. With time, the Vyākaraṇa school accepted both positions and, possibly due to an innovation of Patañjali, began to defend the view that words can denote either particulars or universals depending on the context and the need to emphasize one aspect over the other.^7^

The earliest documented works of the Nyāya tradition also adopt a similar approach to the question. Both the *Nyāyasūtra* and Vātsyāyana’s *Bhāṣya* thereon discuss the notion of universals in connection with the problem of word-meaning. Towards the end of a section dedicated to exploring the issue, Gautama defines the universal as “the cause of the idea of generality,”^8^ specifically, as the element that allows one to “unify certain things [belonging to a given class].” Vātsyāyana comments on this *sūtra*, highlighting that a universal property, unlike a generic similarity, can individuate a class. Universals unify the particulars exemplifying them while simultaneously distinguishing them from particulars lacking the property in question:

*yā samānāṃ buddhiṃ prasūte bhinneṣv adhikaraṇeṣu, yayā ca bahūnītaretarato na vyāvartante, yo ’rtho ’nekatra pratyayānuvṛttinimittam, tat sāmānyam, yac ca keṣāñcid abhedaṃ karoti, kutaścid bhedam, tat sāmānyaviśeṣo jātir iti* |

[The universal] that brings forth a similar cognition in relation to different substrata, that by force of which many objects are not distinguished from one another, that meaning which causes the recurrence of a cognition concerning different [particulars], that is a generality. That which unifies certain things while distinguishing them from others is a particular [type of] generality, the “universal.”^9^

A similar discussion occurs in contemporaneous Vaiśeṣika works. As expected from a school primarily focused on classifying reality, the first ontological characterization of universals appears within this tradition. The *Vaiśeṣikasūtra*s, the foundational text of the school, include “universal” among the six fundamental categories (*padārtha*) of reality. However, reconstructing the early phase of Vaiśeṣika is notoriously challenging. While certain parts of the *Vaiśeṣikasūtra*s can be traced back to the first millennium BCE, other sections are believed to be later additions, and the final redaction of the text likely occurred in the early centuries CE. This affects the matter at hand. There are indications that the *sūtra* (1.1.4) listing the six categories may be a later interpolation, as it is absent in several ancient commentaries.^10^ Scholars have hypothesized that the system originally had only three categories (substance, quality, motion), and the subsequent three (universal, particularity, inherence) were added later. Although the exact timing of this development is unknown, this may explain the absence of 1.1.4 in older sources. By the time of Praśastapāda (second half of the sixth century CE), arguably the most influential ancient commentator on the *Vaiśeṣikasūtra*s, the acceptance of six categories is more or less definitive. In his *Padārthadharmasaṃgraha*, we find the earliest ontological definitions of universals in Indian philosophical literature:

*sāmānyaṃ dvividhaṃ param aparaṃ cānuvṛttipratyayakāraṇam* | *tatra paraṃ sattā mahāviṣayatvāt* | *sā cānuvṛtter eva hetutvāt sāmānyam eva* | *dravyatvādy aparam alpaviṣayatvāt* | *tac ca vyāvṛtter api hetutvāt sāmānyaṃ sadviśeṣākhyām api labhate* |^11^

Generality is of two kinds—ultimate and non-ultimate—and is the cause of the notion of recurrence/similarity. Of these two, ultimate [generality] is existence, because it has a vast domain. [Existence] is indeed a generality, as it is the cause of recurrence/similarity. Non-ultimate generality, such as “substance-hood,” etc., has a lesser domain. This [non-ultimate generality], since it is also the cause of differentiation, it is called the individuator of an entity (*sadviśeṣa*).

*sāmānyaṃ dvividhaṃ param aparaṃ ca* | *svaviṣayasarvagatam abhinnātmakam anekavṛtti ekadvibahuṣv ātmasvarūpānugamapratyayakāri svarūpābhedenādhāreṣu prabandhena vartamānam anuvṛttipratyayakāraṇam* |^12^

Generality is of two kinds, ultimate and non-ultimate. It resides in all objects belonging to its class, is undivided and present in many particulars. It produces a cognition conforming to its own nature in the case of one, two, or many objects; being present in the substrata without interruption—since there is no difference [between them] and its own nature—[generality] is the cause of the notion of recurrence/similarity.

In these passages, Praśastapāda reiterates concepts that were already present in Vātsyāyana’s discussion while also introducing new developments. The Vaiśeṣika tradition distinguishes between ultimate and non-ultimate universals. The ultimate universal corresponds to existence itself (*sattā*), it is instantiated by all entities and is the cause of recurrence/similarity. Non-ultimate universals are described as “substance-hood” and other similar properties, encompassing fewer entities. It is noteworthy that while Vaiśeṣika authors generally prefer the term *sāmānya* (translated as “generality”), Nyāya thinkers more commonly use *jāti*. Both traditions, however, share the notion that a universal simultaneously unifies members of a class and differentiates them from entities outside the class. This implies that the concepts of generality/universal (*sāmānya*) and particularity (*viśeṣa*) are inherently relational, and the terms can be appropriately rendered as “genus” and “species,” respectively. Accordingly, a given universal like “pot-ness” is both the generic property shared by all pots and the property that excludes entities from a broader class characterized by “substance-hood.” In other words, “pot-ness” is a genus of pots but also a species of the wider class defined by the property of “being a substance.”

In the second half of the first millennium, thinkers belonging to the Nyāya-Vaiśeṣika tradition explored the issue of universals from various perspectives, addressing problems such as how universals are known, their location, the relationship between the highest universal *sattā* and lower-order universals, and defending realism against criticism primarily voiced by Buddhist nominalist philosophers. However, it appears that the specific problem we are concerned with here, namely, whether there can be a universal of universals and the potential risk of a vicious regress in the realist scheme, was not formalized until a somewhat later stage. Śrīdhara’s commentary on the *Padārthadharmasaṃgraha* (known as the *Nyāyakandalī*), dating back to around the tenth century CE, is perhaps the earliest documented work that suggests the impossibility of construing a universal such as “universal-ness” that would be exemplified in specific universals like “being a pot,” “being red,” or “being to the right of.”^13^ The concept of such a universal implies a vicious regress, a predicament Sanskrit authors typically refer to as *anavasthā*/*anavasthiti*. Apart from these earlier unsystematic references, the first structured examination of the problem of *anavasthā* comes with the work of Udayana, the great logician and epistemologist active in the second half of the tenth century. In his *Kiraṇāvalī*, again a commentary on the *Padārthadharmasaṃgraha*, Udayana presents six conditions that would prevent the application of universals (referred to as *jātibādhaka*, or “blockers of universals”).^14^ One of these conditions is precisely *anavasthā*. The author’s argument is familiar: universals cannot exemplify other universals because that would lead to a vicious regress. However, Udayana advances the discussion by acknowledging that, although ontologically suspect, the concept of a “universal of universals” can be formulated, prompting the question of why this is the case. In response, Udayana introduces a distinction that becomes standard in the Nyāya-Vaiśeṣika works of the second millennium CE,^15^ the distinction between *jāti*, a real, natural (*svābhāvika*) property of an object, and *upādhi*, an adventitious (*āgantuka*) feature attributed to something due to its proximity to something else. The typical example of *upādhi* is the crystal assuming the color of an object nearby. The distinction serves also to address the problem of regress: a universal is a *jāti*, whereas a “universal of universals” is an *upādhi*, or more precisely, an *upādhisāmānya*, a “generality based on an adventitious property” that lacks any ontological equivalence.

## Bhartṛhari on Universals and the Problem of Regress

Centuries before Udayana, Bhartṛhari, in his *Vākyapadīya*, provided an original interpretive framework for analyzing the notion of universals and the potential problem of regress. As a grammarian, it is unsurprising that Bhartṛhari’s analysis originates from the semantic framework discussed in the previous section. His considerations are rooted in the ancient dichotomy that characterizes the debate on the nature of word-meaning in the Pāṇinian tradition: do words denote individual substances or generic properties? Bhartṛhari eventually arrives at an answer that aligns with his tradition, accepting that words can denote both aspects. However, he dedicates the first two chapters of the third book of the *Vākyapadīya*, namely, the *Jātisamuddeśa* and the *Dravyasamuddeśa*, to delve deep into this question. In the *Jātisamuddeśa*, while extensively discussing why one should accept that the meaning of a word is a generic property, Bhartṛhari puts forward a series of compelling considerations about the function of universals. Perhaps his most ground-breaking contribution is the realization that the issue of attribute agreement extends beyond purely ontological questions to encompass aspects of language use. As a matter of fact, words themselves are ontic entities, akin to substances like “cat,” “stick,” or “table.” When we utter different instances of the same word, say “table,” we are uttering a string of sounds that share a common quality: “being the word ‘table’.” This raises the familiar question: how can we explain that the word “table” I am uttering today is the same as the one I uttered yesterday? Or, similarly, why is the word “table” pronounced by another English speaker the same as the one I pronounce? Bhartṛhari’s answer aligns with a realist outlook as he acknowledges the existence of a linguistic universal (*śabdajāti*) as the most plausible explanation for the agreement between different word occurrences.

Generally, the distinction between the linguistic and ontological dimensions and their continuous overlapping is often emphasized in Bhartṛhari’s work. Before becoming relevant to the problem of universals, the notion emerges clearly in the first book of the *Vākyapadīya*. In *Vākyapadīya* 1.55 (= Rau 1.56), Bhartṛhari distinguishes the domains of words and objects by stating that words can reveal both themselves and their referent, much like light does:

*grāhyatvaṃ grāhakatvaṃ ca dve śaktī tejaso yathā* |

*tathaiva sarvaśabdānām ete pṛthag avasthite* ||

Just as light has two capacities, that of cognizing and that of being cognized, exactly in the same way, these two capacities are established as distinguished for all words.

The *Vṛtti* on the *Vākyapadīya*^16^ expands on the analogy, highlighting the ability of words and light to be simultaneously subjects and objects of cognition. This contrasts with physical objects, like pots, which can only be objects of knowledge:

Pots and the like are determined only as being objects of cognitions; therefore, at the moment in which they are grasped, they do not assist, not even slightly, either the sense or the content [of the cognition], as [these latter two are] the [only factors] contributing to the [arising] of the cognition. So, all the senses, which are not cognized, function as the cause for grasping the object to be cognized. On the contrary, light, possessing a nature distinct from that of darkness, becomes the cause of the cognition—in the sense that it contributes to it—[only] when its own form is determined. The word, too, which is differentiated due to its own nature, which is opposed to that of other words and things, conveys the content to be known [only once] its specific nature of word has been comprehended. These two capacities of this [word], that of cognizing and that of being cognized, are always identical but appear as differentiated.^17^

In the following verse, *Vākyapadīya* 1.56 (= Rau 1.57), Bhartṛhari discusses the temporal and logical sequence between these two aspects, stating that words must convey their own form before denoting the meaning:

*viṣayatvam anāpannaiḥ śabdair nārthaḥ prakāśyate* |

*na sattayaiva te ’rthānām agṛhītāḥ prakāśakāḥ* ||

Meaning is not conveyed by words which have not [themselves] become the content [of cognition]. If not grasped, [words] do not convey [their] meanings by their mere existence.

The *Vṛtti* on the *Vākyapadīya* comments:

If words could become subordinate to meanings without having attained the status of primary things to be comprehended, they—whether cognized in relation to their mere existence or not cognized at all—would convey their respective meanings regardless of their content. However, [words] do not convey [meanings] that way. Therefore, in the process through which words become subordinate to meanings, their (that is, the words’) adoption of the form of what is primary is an essential factor.^18^

Bhartṛhari emphasizes here the ambivalent nature of words. They serve as instruments for conveying specific content (*vedaka*, *pramāṇa*) while also being capable of being objects of awareness (*vedya*, *prameya*). This sets them apart from the senses, which are normally not cognized in a perception. By contrast, to understand the meaning of a word, the word must be cognized first. However, Bhartṛhari also underscores that this distinction is merely analytical, as the association between words and meanings is innate and unbreakable. When we hear a word, our immediate and automatic association with a specific meaning makes the analytic examination of signification artificial. That being said, the fact that words are *prameya*, contents of knowledge, indicates that the problem of attribute agreement also exists at the linguistic level.

***Word-Universals and Object-Universals***

By introducing the notions of word-universals (*śabdajāti*) besides word-particulars (*śabda*), Bhartṛhari is expanding the ontological categories beyond the traditional scheme encompassing [object] particulars and [object] universals. The point is to understand if this expansion is justified. To address the question, we can break it down into two related sub-questions: first, is it truly necessary to postulate universals and particulars at the linguistic level? And if so, how are these newly conceptualized categories interconnected? These questions are addressed at the beginning of the *Jātisamuddeśa*. In *Vākyapadīya* 3.1.6, Bhartṛhari reiterates the notion he already had discussed in more general terms in *Vākyapadīya* 1.55–56, that is, words are repeatable entities that exhibit agreement in attributes:

*svā jātiḥ prathamaṃ śabdaiḥ sarvair evābhidhīyate* |

*tato ’rthajātirūpeṣu tadadhyāropakalpanā* |

What is denoted first by all words is just their own universal. After that, there is the conceptualization of an imposition of that [word-universal] on the object-universal’s own form.

This verse specifies that words initially reveal their own linguistic universal, the *śabdajāti*, which is then superimposed on the object-universal (*arthajāti*). The essence of the argument is that, in conceptual analysis, the linguistic aspect is primary relative to the ontological component. That being said, the *Prakīrṇaprakāśa* of Helārāja (tenth century CE), the only extant ancient commentary on the *Vākyapadīya*’s third book, contributes additional insight:

Thus, since [the word-universal] is non-generic, and it is specific [to a word], the relation between it and the word is non-deviant, even when the relation between the word and the object-universal is deviant.^19^

Helārāja clarifies that the relationship between a word-particular and a word-universal differs significantly from that between a word-universal and an object-universal. He emphasizes that the connection between a word-universal and a word-particular is inherent and consistent, while that between a word-universal and an object-universal can vary. This idea can be better understood by resorting to the example of homonyms. Take, for instance, the English word “bat,” which signifies a single word-universal—“being the word ‘bat’.” Yet, its object-universal is ambiguous: the word-particular “bat” can denote either a nocturnal mammal or an instrument used in sports like cricket or baseball. It is true that in the Pāṇinian tradition the relationship between a word-universal and an object-universal is considered fixed (*siddha*, *nitya*), but this “fixedness” implies stability for effective communication, not innateness.

*Vākyapadīya* 3.1.6 also highlights that the superimposition of word-universals upon object-universals is a conceptual process. This is because, in Bhartṛhari’s ultimate view, words and their meanings or referents are fundamentally identical. The temporal articulation of this superimposition is merely analytical. This is why the verse speaks of conceptualization:

Even though the object is instantaneously cognized as essentially identical with the word, [Bhartṛhari], resorting to [the notion] of sequence as explained [above], uses the word “after that” (*tataḥ*): the notion that after the cognition of the [words’] own universal, one conceptualizes this word-universal as imposed on object-universals, such as “cowness” and so forth, is not valid from an absolute perspective; in fact, there is no difference between word and meaning, being meaning a manifestation of the word.^20^

However, the *Prakīrṇaprakāśa* also offers a different explanation for the superimposition being conceptual, an explanation that is perhaps more philosophically interesting:

Alternatively, at the time in which the relation [between words and meanings] is taught—for instance, in the case of a sentence like: “this object is a cow”—since word-universal and object-universal are absolutely different, one conceptualizes an identity [between the word-universal and the object-universal] because their co-referentiality cannot be proven in any other way.^21^

This means that upon encountering an unknown object, one connects the word-universal (the property of “being the word ‘table’”) with the object-universal (“table-hood”), based on the instruction of somebody already knowing the language. Now, although word-universals and object-universals are completely different from one another—for they are instantiated by totally different particulars—they are undeniably co-referential. The only way to explain this co-referentiality is conceptualizing or postulating an identity between the two aspects.

***From Word-Universals to Objects***

The two following verses, *Vākyapadīya* 3.1.7–8, introduce a compelling simile meant to explain the relationship between linguistic universals, object-universals, and particulars. Helārāja regards these verses as addressing an objection based on what we have just considered: why are we supposed to regard object-universal and word-universal as co-referential when they are clearly instantiated by radically different particulars, namely, objects and words? Bhartṛhari replies by saying:

*yathā rakte guṇe tattvaṃ kaṣāye vyapadiśyate* |

*saṃyogisannikarṣāc ca vastrādiṣv api gṛhyate* ||

*tathā śabdārthasaṃbandhāc chabde jātir avasthitā* |

*vyapadeśe ’rthajātīnāṃ jātikāryāya kalpate* ||^22^

Just like the essence of the quality “red” is designated in a red dye (*kaṣāya*), and it is then also grasped in a cloth and so on, because of the proximity with [something] conjoined. In the same way, the universal located in the word, while designating object-universals serves [also] the function of [object] universal, because of the relationship between word and meaning.

The simile involves four elements:

(1) a red quality (*rakta*),

(2) the universal redness (*raktatva*),

(3) a red tint or lacquer (*kaṣāya*), and

(4) a red object like a cloth (*vastra*).

Helārāja tells us that between these elements there is a *saṃyuktasamavetasamavāya* relationship, which means an “inherence in what is inherent in what is in contact.” In this example, the cloth (*vastra*) is in contact (*saṃyoga*) with the red tint (*kaṣāya*), and the universal redness (*raktatva*) inheres in the red tint, but also in a red quality (*rakta*):

“Being red” is the essence of the quality “red,” and it is said to be the generic property “redness” inherent in particular qualities. That [redness], because of the inherence in what is inherent, is designated—that is, serves the purpose of designating [the particular]—in a red dye, [namely,] in a substance that is the substratum for the quality “red.” For it is that substance “red dye,” which is qualified by that [universal “redness”] that is denoted as “red lac.” Cloths and so on are in contact with that [red dye]. There is proximity or contact between the red dye and the conjoined cloth: this is why that “redness” is also cognized in that [cloth]. Therefore, because of the inherence in what is inherent in what is in contact, the designation based on the universal “redness” applies also to cloth, etc., in the sense of “red cloth.”^23^

The second part of the simile, involving the linguistic counterparts, is more complex and has been glossed over by much of the existing literature.^24^ In *Vākyapadīya* 3.1.8, the quality “red” corresponds to a word-particular, such as the word “cow.” “Redness” corresponds to the word-universal “being the word ‘cow’.” The “red tint” corresponds to the object-universal “cow-ness” instantiated by an actual cow. Upon this object-universal, the word-universal “being the word ‘cow’” is superimposed. However, and finally, the very same word-universal can also be imposed upon another object-universal, which is distinct from the original one. This latter step of the simile accounts for the existence in language of figurative meanings, but also proves that, notwithstanding the superimposition, there is a difference between word-universals and object-universals. In this regard, Helārāja discusses the stereotypical sentence “the Vāhīka is an ox,” which figuratively means that a person from a certain region (Punjab?) is dull-witted and slow as an ox. Here, we have the quality expressed by the word “cow” (*gośabda*), the word-universal (*gośabdatva*), a primary substance representing a particular instantiating an ontic universal (“being an [actual] ox,” *gotva*), and a second substance (the Vāhīka person) related to the first metaphorically.^25^ The *saṃyuktasamavetasamavāya* relationship is clearly visible here. The second substance (the Vāhīka) is in contact with the first substance (the cow instantiating “cowness,” *gotva*). This contact is metaphorical and not innate. The universal of the first substance does not deviate from the word-universal (*gośabdatva*) because of superimposition, and finally, the word-universal inheres in the word “cow” (*gośabda*). Helārāja clarifies this relationship by stating: “The word-particular is first designated as expressed by the word-universal without [reciprocal] distinction; then the object-universal [is designated], and through the latter, the object-particular. This is the factual sequence.” Overall, the analogy aims to explain how a word-universal, such as “being the word cow” denotes external objects, even when the ontic universal of the first substance (*gotva*) is not instantiated in the second substance (*vāhīka*). This is why *Vākyapadīya* 3.1.8 says that word-universal “serves the function of a universal.” Word-universals provide a persistent identity to the objects being denoted even when the object-universals are not instantiated. Therefore, they play a fundamental role in language, not only in specific circumstances like figurative speech but consistently across all instances of language use. They serve as a crucial tool for establishing connections between different cognitive concepts, whether or not those concepts correspond to external objects and their corresponding (ontic) universals.

***The*** **Jātisamuddeśa** ***on the Problem of Regress***

*Vākyapadīya* 3.1.9–10 further delves into examining the disjunction between linguistic and ontic universals by addressing the question of how to form the plural of words denoting universals, all while respecting the principle that universals cannot instantiate themselves:^26^

In the case of an *ekaśeṣa* of words expressing a universal, a word-universal of universals is required. In the case of *śabdajātayaḥ*, that [word-universal] is exemplified in word-universals. The universal exemplified in words expressing universals has a nature that differs from that of words, but [still] it occurs without transgressing its nature of being a word-universal.^27^

When we form the plural of a word, we encompass multiple instances of that same word under a single grammatically plural term. For example, the plural form “trees” implies the inclusion (*ekaśeṣa*, “the remainder of one”) of the word-meanings “tree_1_,” “tree_2_,” and “tree_3_” into a unified word-meaning: trees. In other words, the meaning of the word “trees” refers to the universal property shared by each individual “tree.” While this process is typically straightforward, it becomes problematic when dealing with words that already express a universal property. Bhartṛhari examines this scenario in the case of the plural form of the word *jāti*, namely, *jātayaḥ*. One might assume that the plural of the word *jāti* expresses the word-universal (we have just seen that the plural implies a universal feature) of a universal itself (as *jāti* means “universal”). However, Bhartṛhari clarifies that this case has no ontological commitment. The term *jātitva* does not correspond to a specific entity in the real world. Nonetheless, the plural of *jāti* is possible because of the existence of the word-universal *jātiśabdatva*. This word-universal serves as a linguistic substitute whenever an object-universal is not applicable, as is the situation here. This universal is exclusively confined to the realm of language.

Taking the discussion further, one can examine the word *śabdajāti* itself, which, naturally, has a plural form and might give the impression of instantiating another universal, namely, *śabdajātitva*. However, Bhartṛhari explicitly refutes this notion, asserting that even in the case of a supposed *śabdajātitva*, what is involved is always the first-order level *śabdajāti*. To clarify this, the following scheme can be presented:

*First-order series*: The plural *jātaya* of the word *jāti* is possible due to *jātiśabdajāti* (the universal of the word *jāti*), yet this universal ultimately represents nothing more than a linguistic universal, *śabdajāti.*

*Second-order series*: One can still employ the just-established word-universal, *śabdajāti*, and assert that its plural form *śabdajātayāḥ* is possible due to the universal *śabdajātijāti*.

If one allows this, the charge of infinite regress becomes serious. However, Bhartṛhari argues that the final elements of the two series, *jātiśabdajāti* and *śabdajātijāti*, are the same. Since their respective exemplifying items, *jāti* and *śabdajāti*, are both words, they are, in the end, both *śabdajāti*, that is, linguistic universals. Therefore, the regress never occurs. No matter how far one may continue the instantiation-regress game, this remains a pseudo-regress that pertains solely to the linguistic aspect of universals and has no real-world implications. Ontologically, the only actual universal that exists is the first-order one. Helārāja rounds the discussion off by stating that the problem of regress does not pertain to language.^28^

This conclusion is further explored and taken to extremes in the subsequent verse, *Vākyapadīya* 3.1.11. Bhartṛhari introduces here an alternative explanation of the relationship between words and object-universals, leaving aside the concept of imposition (*adhyāsa*) and opting for a more straightforward approach. In this second option, words directly denote object-universals without the need for intervening word-universals.^29^ This shift may sound weird, considering the extensive efforts made to define and refine the notion of linguistic universals. However, there is a reason for this unexpected move. Helārāja suggests that the concept of *adhyāsa* presents significant difficulties. In cases where the word-universal serves as an object-universal, as in the example of the plural form *jātayaḥ*, there is no superimposition between a word-universal and an object-universal. Why? Simply because there is no object-universal corresponding to the word *jātayaḥ*. But if there is no object-universal, how can we talk about superimposition? On what the word-universal would be superimposed?^30^ One available strategy would be to postulate an object-universal for the word *jātayaḥ* as well. However, this would really be an (ontic) universal of universals, thereby facing the predicament of regress. According to Helārāja’s interpretation, Bhartṛhari shifts to the thesis that words directly denote universals to avoid the problems implied in the notion of superimposition. In the end, his definitive position consists of drawing a clear line between how the Vaiśeṣikas (explicitly brought into the discussion in the *Prakīrṇaprakāśa* on 3.1.11) conceive universals and how the grammarians understand them.^31^ From the grammarian’s perspective, the fundamental point is that a specific sequence of sounds elicits a particular cognition that may or may not be related to a specific external object.^32^ In this perspective, the essence of an object is less about its intrinsic ontological nature and more about the role its corresponding word plays within a specific linguistic context. This means that if what is universalized in a sentence is an attribute (*guṇa*), the grammarian is allowed to describe this attribute as a “universal,” regardless of whether such a definition is accurate from an ontological standpoint. The same principle applies to the notion of action (*kriyā*) or substance (*dravya*). The conclusion is that, from a linguistic perspective, it is perfectly fine to engage in discussions about universals of universals or, say, particulars of particulars.^33^

This development, wherein a universal is perceived as a function rather than an entity with a well-defined ontic status, may appear to challenge Bhartṛhari’s allegiance to realism. However, it would be an exaggeration to assert that Bhartṛhari denies the existence of genuine, ontic universals, as he fundamentally maintains a realist stance, which he reiterates often in his work.^34^ In my understanding, Bhartṛhari is rather advising caution, pointing out that even if most of the time word-universals correspond to object-universals, sometimes they do not (as is evident in instances such as the formation of the plural form of the word *jāti*, or metaphorical usage).

## A Different Solution to the Problem of Regress?

Let us finally consider whether Bhartṛhari’s idea that higher-order steps in the instantiation of universals are exclusively linguistic can provide a different solution to the issue of regress. To illustrate the point, let us focus on the following example again involving a subject-predicate sentence:

(1) The table is brown.

This statement is true if:

(2) The table is an instantiation of [brownness].

But (2) can only be true if:

(3) The table is an instantiation of an [instantiation of brownness].

In this way, we apparently encounter a vicious regress. Bhartṛhari, however, would argue that here there is no vicious regress at all. The first-order universal, “brownness” is a word-universal that is inherently related to an object-universal, which is instantiated by brown particular objects, a table here. The situation changes when we consider [instantiation of brownness], which can be seen as a second-order universal. Although [brownness] can indeed function as the instantiating particular for the universal [instantiation of brownness], [instantiation of brownness] itself is merely a word-universal. It lacks an ontological counterpart.

In a broader sense, Bhartṛhari’s suggestion to distinguish between object-universals and word-universals offers an alternative way to refute the charge of regress often directed towards the realist framework. According to his view, the possibility of higher-order instantiations is not rejected. In fact, the series of instantiations can be potentially infinite. However, the instantiated universals from the second-order level and beyond are only word-universals or linguistic conceptualizations, lacking any ontological equivalence. Bhartṛhari’s solution to the problem of regress aligns, therefore, with the argument that there may be a regress within the realist framework, but this regress is not vicious. His originality lies in emphasizing the role played by language in the process. From a linguistic perspective, higher-order instantiations are possible, but only first-order universal properties are ontologically exemplified.

## Conclusion

This paper has aimed to demonstrate that Bhartṛhari approached the problem of universals in an original manner. Building upon the widely attested Indian perspective that the issue of attribute agreement is also relevant to semantics, Bhartṛhari introduced a significant distinction between word-universals and object-universals. Although this distinction expands the number of fundamental categories, it offers an alternative solution to the problem of regress—a criticism often leveled against realism. From a historical standpoint, it is noteworthy that Bhartṛhari was among the first authors to address the issue of regress of universals explicitly. His discussion seems to precede any surviving works of the Nyāya-Vaiśeṣika tradition. However, whether Bhartṛhari was really the first to conceptualize the problem of regress is hard to tell, as he may have been familiar with earlier, now lost, Nyāya-Vaiśeṣika works.^35^

## Footnotes

1 For the sake of simplicity, in this paper, I am using “realism” in reference to the Platonic version of the theory. Of course, many forms of realism have been devised by Euro-American philosophers over time, with the most notable opposition to the Platonic version being that of Aristotle, who accepted the existence of universals but argued that they could not exist separately from instantiating particulars.

2 The charge of regress of universals is as old as the notion of universals itself and is already prefigured in Plato’s *Parmenides*. For a theoretical introduction to the problem of regress of universals, see Loux 2002: 17–45. Among contemporary discussions of the issue, one could cite Strawson 1959, Donagan 1963, Loux 1978, and Armstrong 1989.

3 The argument is associated with F. H. Bradley and is today known as Bradley’s regress. For its original formulation, see Bradley 1893: 19–21.

4 The most comprehensive study of universals in Indian philosophy remains Dravid 1972. For a specific examination of the way the Nyāya-Vaiśeṣika tradition dealt with the issue, see Chakrabarti 1975. The most important advocates of nominalism were the Buddhists. The logico-epistemological school of Dignāga and Dharmakīrti introduced a revolutionary semantic theory—the *apoha* theory—which works also as a nominalist explanation of the problem of attribute agreement.

5 On the complex relationship between the theories of universals in Vyākaraṇa and Vaiśeṣika, see also Lysenko 2007.

6 *ākṛtyabhidhānād vaikaṃ vibhaktau vājapyāyanaḥ* (*Vārttika* 35 on A 1.2.64; Kielhorn 1880: 242); *dravyābhidhānaṃ vyāḍiḥ* (*Vārttika* 45 on A 1.2.64; Kielhorn 1880: 244). We do not know anything about these two grammarians, who must have lived between Pāṇini and Kātyāna’s times, sometime around the fourth century.

7 *na hy ākṛtipadārthikasya dravyaṃ na padārtho dravyapadārthikasya vākṛtir na padārthaḥ* | *ubhayor ubhayaṃ padārthaḥ* | *kasyacit tu kiṃcit pradhānabhūtaṃ kiṃcid guṇabhūtam* | *ākṛtipadārthikasyākṛtiḥ pradhānabhūtā dravyaṃ guṇabhūtam* | *dravyapadārthikasya dravyaṃ pradhānabhūtam ākṛtir guṇabhūtā* | “Actually, for the supporter of the notion that word-meaning is a generic form, it is not the case that [also a particular] substance [cannot be] the word-meaning. Or, alternatively, for the supporter of the notion that word-meaning is a [particular] substance, it is not the case that the generic form [cannot be] the word-meaning. For both [positions], both [particular and universals] [can be] word-meanings. Still, to some, one [position] is primary and the other subordinate. For the supporter of word-meaning being a generic form, the generic form is primary and the [particular] substance is subordinate. For those supporting word-meaning being the [particular] substance, the [particular] substance is primary and the generic form is subordinate” (Kielhorn 1880: 242–43). All translations are mine unless otherwise stated.

8 See *Nyāyasūtra* 2.2.69: *samānaprasavātmikā jātiḥ* (Thakur 1997)

9 See *Nyāyabhāṣya* on *Nyāyasūtra* 2.2.69 (Thakur 1997: 133–34).

10 See Halbfass 1992: 75.

11 See *Padārthadharmasaṃgraha* (Bronkhorst and Ramseier 1994: 2).

12 See *Padārthadharmasaṃgraha* (Bronkhorst and Ramseier 1994: 81).

13 See *Nyāyakandalī* (Dvivedin 1984: 314).

14 See *Kiraṇāvalī* (Sārvabhouma 1989: 148–52).

15 See Potter 1977: 136. The conception of *upādhi* is clearly interpreted as a later innovation in the history of Nyāya, see Pellegrini 2016. Hints at the notion are also present in Śrīdhara’s work.

16 This commentary on the first two books of the *Vākyapadīya* is traditionally ascribed to Bhartṛhari. Although some contemporary scholars question this attribution, I am inclined to favor the traditional view of Bhartṛhari’s authorship.

17 *Vṛtti* on the *Vākyapadīya* on 1.55 (= Rau 1.56): *ghaṭādayo hi grāhyatvenaiva vyavasthitā iti ghaṭādīnāṃ grahaṇakāle naiva kāṃcid upalabdhisahakāritvenendriyasya viṣayasya vā svalpām apy anugrahamātrāṃ kurvanti* | *tathā grāhyatvam apratipadyamānāny eva sarvendriyāṇi grāhyasya viṣayasya grahaṇanimittatvāya kalpante* | *tejas tu tamorūpavirodhinā rūpabhedena yuktam avadhāryamāṇasvarūpam anugrāhakatvenopalabdhau kāranabhāvaṃ pratipadyate* | *śabdo ’pi bhāvāntaravirodhinā śabdāntaravirodhinā ca svarūpeṇāvadhriyamānabhedaḥ parigṛhītaviśeṣasabdasvarūpaḥ pratyāyyam arthaṃ prakāśayati* | *te cāsya pratipādyapratipādakasaktī nityam ātmabhūte pṛthaktveneva pratyavabhāsete* | (Iyer 1966: 115, lines 5–10; 116, lines 1–2).

18 *yadi grāhyatvena śeṣibhāvam apratipannāḥ śabdāḥ śeṣatvam artheṣu pratipadyeran te sattāsaṃbandhamātreṇopalabdhāś ca anupalabdhāś ca viṣayatvam anapekṣamānāḥ svaṃ svam arthaṃ pratyāyayeyur* | *na tu pratyāyayanti. tasmād aṅgabhūta eṣām artheṣu guṇabhāvāpattau prādhānyamātrārūpeṇa saṃsparśa iti* | (Iyer 1966: 116, lines 6–9).

19 *evaṃ cāsadhāraṇatvena viśeṣaṇāt tayā saṃbandhāvyabhicāraḥ śabdasyārthajātyā saṃbandhavyabhicāre ’pīti* | See *Prakīrṇaprakāśa* on *Vākyapadīya* 3.1.6 (Iyer 1963: 16, lines 16–17).

20 *arthasya jhaṭity eva śabdasvarūpābhedenāvabodhe ’pi yathāpratipāditakramāśrayeṇa tataḥ svajātipratyāyanād anantaram arthajātināṃ gotvādīnām ātmasu tasyāḥ śabdajāteḥ samāropasya kalpanā paramārthaḥ, śabdavivartatvenārthasya śabdāt tattvato bhedābhavāt* | (Iyer 1963: 17, lines 11–14).

21 *yad vā saṃbandhavyutpattikāle gaur ayam artha ity arthajātyā śabdajāter atyantabhedāt sāmānādhikaraṇyānyathānupapattyā abhedādhyaropaḥ kalpyate* | *Prakīrṇaprakāśa* on *Vākyapadīya* 3.1.6 (Iyer 1963: 17, lines 15–16).

22 Iyer 1963: 19, lines 3–6.

23 *tasya raktasya guṇasya bhāvo raktatvaṃ lauhityasāmānyaṃ guṇavyaktisamavetam ucyate* | *tatsamavetasamavāyāt kaṣāye raktaguṇādhāre dravye vyapadiśyate vyapadeśāya kalpate* | *tadviśeṣaṇabhūtaṃ hi tatkaṣāyadravyam abhidhīyate lohitā lakṣeti* | *tena saṃyogino vastrādayaḥ tayoḥ saṃyoginoḥ kaṣāyavastrayoḥ saṃnikarṣaḥ saṃbhedaḥ, tasmān nimittāt tatrāpi tallauhityaṃ gṛhyate* | *saṃyuktasamavetasamavāyād vastrādiṣv api lauhityanimitto vyapadeśaḥ, lohitaṃ vastram ity arthaḥ* (Iyer 1963: 19, lines 7–12).

24 Iyer (1971) and Herzberger (1986) have correctly explained the relation occurring at the ontic level described in *Vākyapadīya* 3.1.7. Still, they did not address the troublesome nature of the second item of the comparison in *Vākyapadīya* 3.1.8ab (Iyer 1971: 11–12; Herzberger 1986: 85–94). Kobayashi (2009) gives a fairly correct interpretation of this verse but without delving into the details of the analogy. To my knowledge, Benjamin’s (2019: 187–92) unpublished doctoral dissertation is the only work that takes *Vākyapadīya* 3.1.8 into proper consideration, providing a thorough analysis of its meaning.

25 The notion of *saṃyuktasamavetasamavāya* is not Helārāja’s interpretation but is attested also in Bhartṛhari’s *Māhabhāṣyaṭīkā*, as shown in Benjamin (2019: 191–92): *nīlaśabdo ‘rthāntaranirapekṣo guṇasāmānyas tadrūpaṃ pratilabhate* | *tatra pratilabdhasvarūpas tadadhiṣṭhāno yadā guṇavacano nīlasya sāhacāryāt nīlatvena vyāveśaṃ taddharmatām anubhavati* | *ekayogakṣematām āpadyate vibhāgābhāvād ākṛtyabhibhūta eva san guṇapratyayam ādadhāti* | *yathopāśrayavaśāt sphaṭikaṃ tadguṇam upalabhyate, evaṃ dravyam apy ayam abhidadhan nīlaśabdaḥ svābhidheyasya nīlatvasya ya āśrayo guṇas tasyādhāro dravyam iti samavetasamavāyād vyavahito’pi guṇena tadadhiṣṭhāna eva dravye pratyayam ādadhāti* | *tathā dravyāntareṇāpi rakte vastre* | *saṃyuktasamavetasamavāyād eva pāramparyeṇa yāva [d] dvayo raktayor vastayor madhye śuklaṃ vastraṃ tadguṇam upalabhyate iti* | *evaṃ gaur ity apy ayaṃ śabdo gotve pratilabhya svarūpaṃ tadadhiṣṭhāna eva dravye pravartate* | The word “blue” does not depend on any other object, expresses a general quality and assumes the form of that [quality]. Concerning this, when [the word “blue”], expressing a quality, has acquired the nature of that general quality and becomes its substratum, it attains a state of complete pervasion, that is, an identity of nature, with the [universal] “blue-ness” because this latter is associated with [the particular] “blue.” [Hence], [the particular word] attains equal footing with [the universal]. Since the [two, that is, the particular “blue” and the universal “blueness”] are not distinguished, when the notion of [universal] form is suppressed, [the word] conveys that of quality. Just like a crystal is perceived to have a quality because of something close to it, in the same way when this word “blue” denotes a substance, the quality which is the substratum of the universal “blueness” denoted by the word has as its [own] substratum [that] substance. Even though [the word] is not contiguous [to the substance]—on the basis of the relationship of “inherence in what inheres,” [the word], through [the denotation of] quality, conveys also the notion of substance, this indeed being the substratum of that [quality]. Thus, in the case of a red cloth the notion of substance is conveyed by another substance. On the basis of a relationship of “inherence in what is inherent in what is in contact,” as long as a white cloth is put between two red clothes, it is perceived as having their qualities. Similarly, also this word “cow,” having assumed its proper nature of “cowness,” it is also applied to substance because that is the substratum of that [“cowness”]. See *Mahābhāṣyaṭīkā* (Bronkhorst 1987: 15).

26 See also the discussion in Bronkhorst 1991.

27 *Vākyapadīya* 3.1.9–10: *jātiśabdaikaśeṣe sā jātīnāṃ jātir iṣyate* | *śabdajātaya ity atra tajjātiś śabdajātiṣu* || *yā śabdajātiśabdeṣu śabdebhyo bhinnalakṣaṇā* | *jātiḥ sā śabdajātitvam apy atikramya vartate* || (Rau 1977: 106). Iyer 1963 has a different reading of *Vākyapadīya* 3.1.10a: *yā śabdajātiḥ śabdeśu*.

28 *Prakīrṇaprakāśa* on *Vākyapadīya* 3.1.10: *na prayogānavasthāpattir ity abhiprayaḥ* “The [overall] sense is that the fault of regress does not concern [language] use” (Iyer 1963: 23, line 4).

29 Helārāja calls this new position of Bhartṛhari “bold,” “audacious” (*prauḍha*). See *Prakīrṇaprakāśa* introducing 3.1.11 (Iyer 1963: 23, line 7).

30 *Prakīrṇaprakāśa* on *Vākyapadīya* 3.1.11: *astu śabdajāter arthajātināṃ jātikāryaṃ saṃbandhavyutpattis tu na ghaṭate* | *yatra hi saṃketas tatra śabadjātir adhyasyate* | *sa eva tv anekatvād arthajātināṃ sāmānyāntaram antareṇa na saṃbhavatīti* | “If we admit that the word-universals serve the function of universals for object-universal, then in that case there will not be the comprehension of the relation [between word and meaning]. For, whenever there is a convention, the word-universal [has to be] imposed [on the object-universal]. But such a convention is [in this case] impossible without postulating another universal, because of the multiplicity of object-universals” (Iyer 1963: 23, lines 10–12).

31 *Prakīrṇaprakāśa* on *Vākyapadīya* 3.1.11: *vaiśeṣikādināṃ bhavantu niḥsāmānyāni sāmānyāni* | *tāni hi vyaktiṣv anvayapratyayāvaseyāni paropādhirūpāṇi svatantravyaktivad idantāvabhāsitvābhāvāt sāmānyāntareṇa nopādhīyante* | “According to the Vaiśeṣika and other [thinkers], there are universals without [further] universals. For, these universals are ascertained by perceiving an association between particulars, and [because] they delimit these particulars from other particulars. They are not delimited by another universal because they do not exist as ‘being *x*’ (*idantā*) as an independent particular does” (Iyer 1963: 24, lines 1–3).

32 *Prakīrṇaprakāśa* on *Vākyapadīya* 3.1.11: *vaiyakaraṇāṃ śabdārtho ’rtha ity abhyupeyatām anvayirūpāvacchedena śābdasya pratyayasyotpatter jātiṣv api jātir aviruddhābhyupagamyā* | *etad eva hi vyaktiṣv api jātyabhyupagame nimittam ity atrāpi tathābhyupagame kaḥ pradveṣaḥ* | *pratyakṣā api ca padārthāḥ svakāryaṃ vijñānādikaṃ kurvantaḥ santīti vyavahriyante, kiṃ punaḥ śabdavācyāḥ padārthā iti sāmānyavacanād dravyādayo ’pi śabdavācyatvenaiva lakṣyante* | “Since the grammarians hold that the object is a linguistic content, the universal of universals is accepted as non-contradictory, because the production of a linguistic cognition occurs due to the determination of a homogeneity [between particulars]. In fact, that being the reason for accepting that there are universals for particulars, what is the problem with accepting the same [in the case of universals of universals]? Actually, perceivable objects, too, are defined as existent in so far as they perform their function, which is that of causing knowledge, etc.; so why [would it not be the same for] objects expressed by words? Since [words] express a universal, even substances, etc., are recognized as being expressible through words only” (Iyer 1963: 24, lines 4–8). And also: *etac ca laukikavyavahārānuguṇyena śāstre asmin vyutpādyate* | *śāstrāntaraprasiddhā hi vyavasthā lokaviruddha* | *loke hi gavi śṛṅgam, vṛkṣe śākhā it vyavahāraḥ* | *tathaiva ca vyākaraṇe apy ādhārasaptamī* | *śāstrāntare tv avayaveṣv avayavīti śṛṅge gauḥ, śākhāyāṃ vṛkṣaḥ, iti syāt* | *itthaṃ ca śabdābhidheyasyehārthatvāt sarve jātyabhidhayinaḥ, yasmāc chabdavyāpāreṇa padārthā lakṣyante* | “In this discipline, [all] this is explained in conformity with common usage since what is recognized in other disciplines is in conflict with common practice. In normal speech, we say that the ‘horn resides in the cow’ or the ‘branch resides in the tree.’ This is so because, in grammar, this is a locative indicating the locus. However, in other disciplines, it is [believed] that the whole resides in the parts, so ‘the cow resides in the horns’ and ‘the tree resides in the branch.’ In this manner, since here [in grammar] an object is what is denoted by words, all words [that is, not only those expressing generality] denote a universal, as objects are defined based on the function of words” (Iyer 1963: 25, lines 1–5).

33 Bhartṛhari himself restates the notion in a later verse of the third *kāṇḍa*. See *Vākyapadīya* 3.11.11: *sāmānyeṣv api sāmānyaṃ viśeṣeṣu viśiṣṭatā* | *saṃkhyāsu saṃkhyā liṅgeṣu liṅgam evaṃ prakalpate* || “In this way, one can conceptualize a generality for generalities, a particularity for particularities, a grammatical number for grammatical numbers, a grammatical gender for grammatical genders.”

34 The *Prakīrṇaprakāśa* on *Vākyapadīya* 3.1.7–8 is rather explicit in regarding Bhartṛhari’s discussion of universals as a form of realism. In a realist perspective, the object-universal exists independently from the word-universal and, consequently, also before the process of language learning begins: *svābhāvikaś cāyam arthapratyāyane śabdānām abhedasaṃbandho ‘bhyupāya iti pratipādyo ’pi tathaiva pratipadyate* | *na hi puruṣādḥinam etat* | *anadau saṃsāre ’nenaiva prakāreṇa saṃbandhavyutpatteḥ ko ’tra niyato ’dhyāropayitā kalpyatām* | *avyutpannasaṃketasyāpi cābhinnapratyayotpatter arthajātir apy astīti, na śabdajātir evādhyāropitās tv iti vācyam* | “It is agreed that the identity-relation between words and their [activity of] conveying objects is innate: though it is [itself] to be established, [the relationship] also establishes [the object] as such. In fact, this [activity of superimposition] is not dependent on humans. Since in the beginning-less cycle of births and deaths, the learning of the relation [between word and meaning has always taken place] in this way, who is supposed to be the specific creator of the superimposition? Since even for someone who has not learned the convention there is the coming into being of a cognition [that does not distinguish the word from the object], also the object-universal exists; it should not be said: ‘Let there be that there is only the word-universal which is superimposed upon [something]’” (Iyer 1963: 21, lines 3–6). Moreover: *prāk ca saṃbandhasaṃvedanasamayād arthajātayo bhedenāvabhanty eva* | *avyutpannasaṃketasya bālakasya puro ‘vasthiteṣu sāsnādimatsu piṇḍeṣu [a]bhinnaḥ pratyaya utpadyate tadā vācakasaṃnidhānābhāvād abhinnapratyayahetur arthajātir avadhāryate* | “Object-universals appears to be distinguished before one has an awareness of the relation [between word and meaning]. A child who does not know the convention [yet], has an undifferentiated cognition in relation to objects that have previously been ascertained as possessing a dewlap, etc.; then, because there is no expressing word available, what is determined [by the knower, that is, the child] it is the object-universal which is the cause of a unitary cognition” (Iyer 1963: 21, lines 9–12).

35 See Potter (1977: 238–39) for an overview of pre-Praśastapāda works that Bhartṛhari may have known.
